# Chemotherapy Agents Alter Plasma Lipids in Breast Cancer Patients and Show Differential Effects on Lipid Metabolism Genes in Liver Cells

**DOI:** 10.1371/journal.pone.0148049

**Published:** 2016-01-25

**Authors:** Monika Sharma, Jo Tuaine, Blair McLaren, Debra L. Waters, Katherine Black, Lynnette M. Jones, Sally P. A. McCormick

**Affiliations:** 1 Department of Biochemistry, University of Otago, Dunedin, New Zealand; 2 Southern Blood and Cancer Service, Dunedin Hospital, Dunedin, New Zealand; 3 Department of Medicine, University of Otago, Dunedin, New Zealand; 4 Department of Human Nutrition, University of Otago, Dunedin, New Zealand; 5 School of Physical Education, Sport and Exercise Sciences, University of Otago, Dunedin, New Zealand; University of Milano, ITALY

## Abstract

Cardiovascular complications have emerged as a major concern for cancer patients. Many chemotherapy agents are cardiotoxic and some appear to also alter lipid profiles, although the mechanism for this is unknown. We studied plasma lipid levels in 12 breast cancer patients throughout their chemotherapy. Patients received either four cycles of doxorubicin and cyclophosphamide followed by weekly paclitaxel or three cycles of epirubicin, cyclophosphamide and 5’-fluorouracil followed by three cycles of docetaxel. Patients demonstrated a significant reduction (0.32 mmol/L) in high density lipoprotein cholesterol (HDL-C) and apolipoprotein A1 (apoA1) levels (0.18 g/L) and an elevation in apolipoprotein B (apoB) levels (0.15 g/L) after treatment. Investigation of the individual chemotherapy agents for their effect on genes involved in lipoprotein metabolism in liver cells showed that doxorubicin decreased ATP binding cassette transporter A1 (ABCA1) via a downregulation of the peroxisomal proliferator activated receptor γ (PPARγ) and liver X receptor α (LXRα) transcription factors. In contrast, ABCA1 levels were not affected by cyclophosphamide or paclitaxel. Likewise, apoA1 levels were reduced by doxorubicin and remained unaffected by cyclophosphamide and paclitaxel. Doxorubicin and paclitaxel both increased apoB protein levels and paclitaxel also decreased low density lipoprotein receptor (LDLR) protein levels. These findings correlate with the observed reduction in HDL-C and apoA1 and increase in apoB levels seen in these patients. The unfavourable lipid profiles produced by some chemotherapy agents may be detrimental in the longer term to cancer patients, especially those already at risk of cardiovascular disease (CVD). This knowledge may be useful in tailoring effective follow-up care plans for cancer survivors.

## Introduction

Cancer is a common cause of death worldwide [1]. Much progress has been made to reduce the morbidity posed by cancer with the increased efficacy of chemotherapy in the past 30 years. Cancer survival rates have dramatically improved over this period [2]. However, chemotherapy-related complications may affect survival; the most significant being the effects of chemotherapy on cardiovascular health [3]. Indeed, cardiovascular disease (CVD) is the most common cause of death, next to the cancer itself, in many forms of solid tumours including breast cancer [4, 5]. A recent study showed that CVD competes with breast cancer as the leading cause of death in women diagnosed with breast cancer, particularly in older women and in those with an early stage diagnosis [6].

A common chemotherapy regimen for breast cancer patients includes a combination of doxorubicin and cyclophosphamide followed by paclitaxel [7]. Doxorubicin is an anthracycline that is well known to be cardiotoxic [8]. This is largely due to its ability to invoke reactive oxygen species production and lipid peroxidation and to cause an excessive release of cytochrome c, which induces apoptosis [8]. Cyclophosphamide has been reported to be cardiotoxic at high doses and has been shown to amplify doxorubicin-induced cardiomyopathy [9]. The cardiotoxicity of the taxane, paclitaxel has been debated in the literature with evidence for [10] and against [11] an additive effect on doxorubicin-induced cardiomyopathy.

Although cardiotoxicity plays a large role in the increased risk of CVD in breast cancer patients through significant alterations in heart function [5], it is also likely that chemotherapy agents may alter other significant CVD risk factors. There have been reports of chemotherapy agents affecting plasma lipid levels. An increase in total cholesterol and low density lipoprotein (LDL) cholesterol was observed in 30 chronic myeloid leukaemia patients after chemotherapy [12]. A study of 70 breast cancer patients showed both high density lipoprotein (HDL) and LDL cholesterol levels to be decreased after chemotherapy with 5’-fluorouracil, methotrexate, and cyclophosphamide [13]. The mechanisms underlying changes in plasma lipid levels with chemotherapy are unknown, but likely to be agent-specific.

Here we aimed to investigate the longitudinal effect of chemotherapy on lipids in the same group of patients by monitoring the serum lipid profiles of 12 breast cancer patients throughout their multi-agent chemotherapy treatments. We show that the chemotherapy treatment produced significant alterations in plasma lipids and apolipoprotein levels creating an unfavourable profile with respect to CVD. Furthermore, we show that individual chemotherapy agents alter the expression levels of key molecules involved in LDL and HDL metabolism in an agent dependent manner, uncovering mechanisms for the alteration in lipid profiles.

## Methods

### Ethics Statement

Ethical approval for this study was obtained from the Lower South Regional Ethics Committee (LRS/10/03/009) and the Southern District Health Board Ethics Committee (Project ID 00626); all participants gave written consent.

### Study Participants

Twelve women (aged between 25 and 65) with newly diagnosed primary breast cancer undergoing multi-agent chemotherapy were recruited over a twelve month period from the Oncology Department at Dunedin Hospital. Participants were excluded from the study if they had recurrent breast cancer, distant metastases, known thyroid problems or diabetes. The majority of patients were hormone receptor positive, both estrogen receptor (ER) and progesterone receptor (PR). Only two were human epidermal growth factor receptor 2 (HER2) positive. None were on antiestrogen therapy. The 12 patients were in various stages of the disease (2 subjects were at stage IA, 5 at stage IIA, 3 at stage IIB and 2 at stage IIIC according to the American Joint Committee on Cancer (AJCC) classification system). The chemotherapy regimens used in seven women consisted of four, three-weekly treatments with doxorubicin and cyclophosphamide followed by 12 weekly treatments of paclitaxel. Five women received three, three-weekly cycles of epirubicin, cyclophosphamide and 5’-fluorouracil, followed by three, three-weekly cycles of docetaxel. Baseline blood samples were taken 1–3 days before the first chemotherapy treatment was administered, midpoint samples were taken within 1–10 days of the last multi-agent treatment and the final blood sample was taken within 1–10 days of the last single-agent treatment, after an overnight fast.

### Plasma lipid and apolipoprotein measurements

Plasma lipid levels were measured in baseline, midpoint and final samples. Total cholesterol, HDL cholesterol and triglyceride concentrations were determined using enzymatic reagents (Roche, Basel, Switzerland). LDL cholesterol concentrations were calculated using the Friedewald equation [14]. Plasma Lp(a) and apoB concentrations were measured by enzyme-linked immunosorbent assays (ELISA) using apo(a)-specific monoclonal antibody, a5-hrp [15] and apoB-specific monoclonal antibody, 1D1-hrp [16] respectively. ApoA1 concentrations were measured using the APOAT reagent (Roche). Plasma samples were also subjected to Fast Protein Liquid Chromatography (FPLC) on a Superose 6HR 10/30 column (GE Healthcare Bio-Sciences, Uppsala, Sweden) to separate plasma lipoprotein fractions. Separated fractions were measured for cholesterol concentration by enzymatic assay (Roche).

### Cell culture

Hepatocarcinoma (HepG2 cells) were obtained from American Type Culture Collection (Manassas, VA). Cells were maintained in Advanced Dulbecco's Modified Eagle Medium (DMEM) supplemented with 10% foetal bovine serum (Bio International; Auckland, New Zealand), 2 mM L-glutamine, 0.25 μg/mL amphotericin B, 100 U/mL penicillin, and 100 μg/mL streptomycin (Invitrogen, Carlsbad, CA) at 37°C in a humidified environment with 5% CO_2_. HepG2 cells were treated with doxorubicin (from 2.5 nM to 25 nM), epirubicin (from 2.5 nM to 25 nM), paclitaxel (from 2.5 nM to 25 nM) and cyclophosphamide (from 1 μg/ml to 100 μg/ml) individually for 24 hours in DMEM. Cell viability was checked using the Trypan blue exclusion method [17] and showed that >90% of the cells remained viable after the various treatments.

### Quantitative RT-PCR

Total RNA was extracted from treated HepG2 cells using TRIzol^®^ (Invitrogen, Carlsbad, CA). DNase treatment followed by cDNA synthesis was performed using Quantitect reverse transcription kit (Qiagen, Venlo, Limburg). Quantitative RT-PCR was performed using KAPA SYBR^®^ FAST Universal 2X qPCR Master Mix (Kapa Biosystems, Wilmington, MA) and cDNA as the template on a LightCycler^®^ 480 (Roche, Basel, Switzerland). Gene expression for ABCA1, PPARγ, LXRα, β-2 microglobulin (β2M) and ribosomal protein L27 (RPL27) was determined using specific primers spanning exonic regions. Expression of each target gene was quantified using Ct values after normalising to the reference gene. The expression of normalising genes RPL27 and β2M were not significantly changed with doxorubicin treatment. Primer sequences are given in S2 Table.

### Western blot

Treated and untreated HepG2 cells were harvested and cell lysates were prepared in RIPA buffer (50 mM Tris-HCl pH-7.8, 150 mM NaCl, 0.1% SDS, 0.5% sodium deoxycholate, 1% Triton X-100) supplemented with complete mini protease inhibitors (Roche). Samples containing 40 μg of protein were resolved on 4% polyacrylamide gels for apoB, 7.5% for ABCA1 and LDLR or on 10% gels for LXRα, PPARγ, apoA1, HMGCOR, actin. Proteins were transferred to nitrocellulose membrane and subject to western blotting with primary antibodies specific for ABCA1 (ab7360), HMGCR (ab174830), PPARγ (ab27649), LDLR (ab30532), LXRα (ab41902) (all from Abcam, Cambridge, England), apoA1 (DAKO, Glostrup, Denmark), actin (A5060 from Sigma, St. Louis, MO) and apoB (1D1, reference 16). The quantification of blots was done using the ImageQuant TL software (Amersham Biosciences, Piscataway, NJ) and protein levels were normalized against actin.

### Cholesterol efflux assay

Cholesterol efflux assays on HepG2 cells were performed as described previously [18]. Cells were seeded in 12 well plates at 2 x 10^5^ cells per well and labelled with 0.5 μCi/ml of [1,2 ^3^H (N)]-cholesterol for 48 hours. Cells were treated with doxorubicin (from 2.5 nM to 25 nM), paclitaxel (from 2.5 nM to 25 nM) and cyclophosphamide (from 1 μg/ml to 100 μg/ml) for 24 hours in serum free DMEM then treated with 20 μg/ml purified lipid-free apoA1 for 2 hours. Tritium decay over 5 minutes in the medium and cell lysates with Optiphase Hisafe II scintillation fluid (Perkin Elmer) were measured as disintegrations per minute (dpm) using a liquid scintillator analyzer (Perkin Elmer, Boston, MA). Cholesterol efflux was calculated using the following equation:

Cholesterol efflux = dpm (media with apoA1) - dpm (media without apoA1) / dpm (cells + media) x 100

### Statistical Analysis

Each parameter in the lipid data was expressed as mean ± SEM. As one midpoint sample was missing for one participant and a final point sample was missing for another, we carried forward the last data point for each of these participants. We used a paired student t-test to compare the effect of anthracycline-containing multi-agent regimens at baseline to midpoint measures. The added effect of the taxane was analysed with a paired student t-test comparing baseline to final measures. In both cases p<0.05 was deemed significant. mRNA, protein and cholesterol efflux data are expressed as mean ± SEM and significance was tested by unpaired student t-test using GraphPad Prism software.

## Results

### Chemotherapy alters HDL, LDL and associated apolipoprotein levels in breast cancer patients

**Fig 1:**
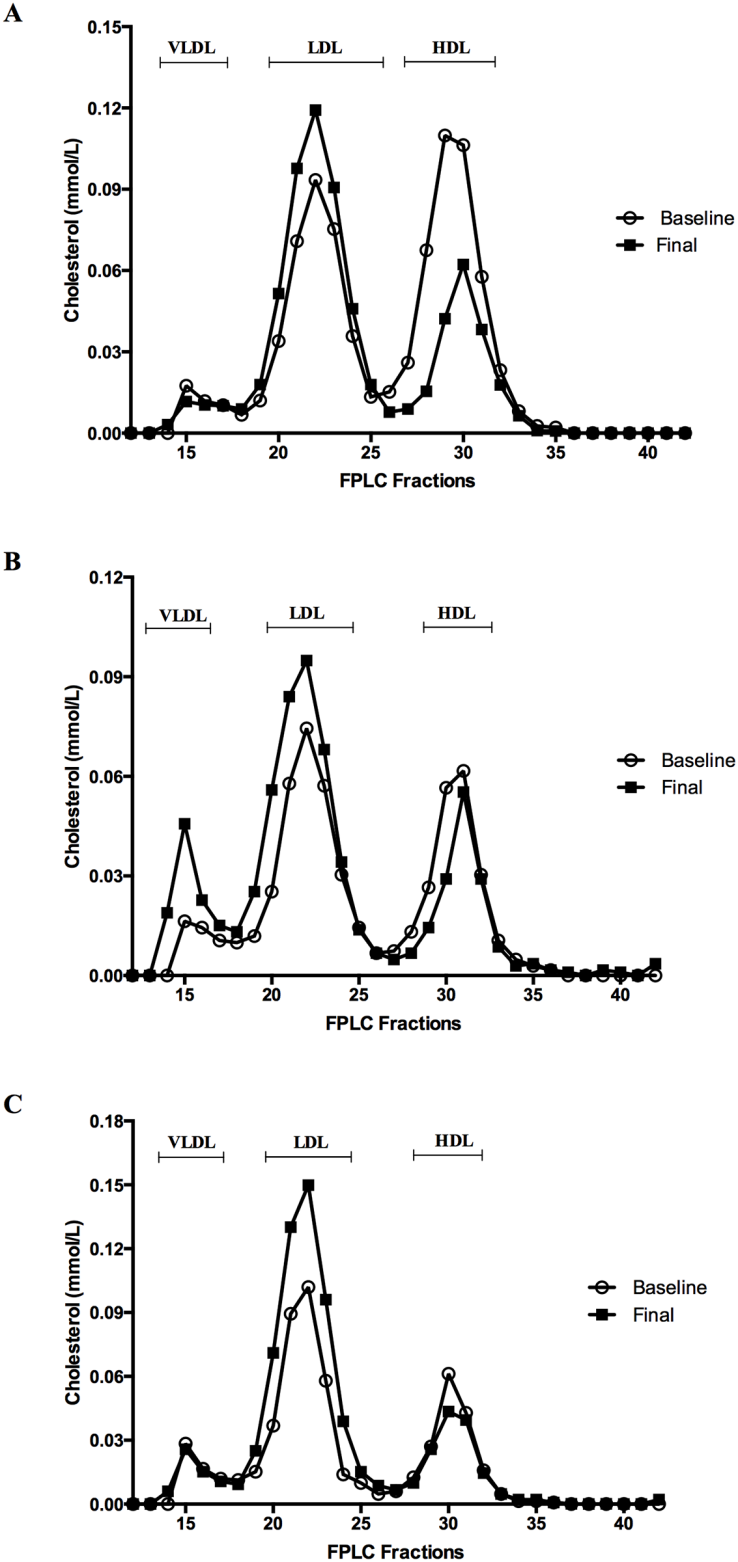
Plasma lipoprotein analysis by FPLC. Plasma samples of breast cancer patients at baseline and final point of treatment were subjected to Fast Protein Liquid Chromatography (FPLC) to fractionate plasma lipoproteins. Cholesterol content in each fraction was measured by enzymatic assay. Representative FPLC profiles of three different breast cancer patient samples are shown (A, B, C).

The plasma lipid levels of each participant before chemotherapy are given in S1 Table. The mean lipid levels in baseline, midpoint and final samples during chemotherapy are given in Table 1. Chemotherapy treatment significantly reduced mean HDL-C levels (0.32 mmol/L) in breast cancer patients from 1.45 mmol/L to 1.28 mmol/ L at midpoint and to 1.13 mmol/L at final point of treatment (p = 0.04 and p = 0.02 respectively, Table 1). The reduction in HDL-C was accompanied by a decrease in apolipoprotein A1 levels (0.18 g/L) from 1.81 g/L at baseline to 1.63 g/L at final point of treatment (p = 0.05, Table 1). Chemotherapy appeared to increase mean LDL-C levels (0.58 mmol/L) from 3.11 mmol/L to 3.57 mmol/L at midpoint and to 3.69 mmol/L after the final point of treatment although only the final point increase reached significance (p = 0.07 and p = 0.04 respectively, Table 1). Accompanying this was a significant increase in apoB levels (0.15 g/L) from 0.97 g/L at baseline to 1.12 g/L at final point (p = 0.001). A correlation analysis of disease stage versus the modulation of lipid levels (Δ mmol/L) showed no correlation (data not shown), likely due to the very low number in each stage. The HDL-C decreasing and the LDL-C increasing effect was apparent on FPLC profiling of lipoprotein fractions from plasma samples taken from breast cancer patients at baseline and final points (Fig 1). There were no significant differences in total cholesterol, triglyceride or Lp(a) levels with chemotherapy treatment (Table 1).

**Table 1:** Mean lipid levels in breast cancer patients.

Serum Lipids	Baseline (mean ± S.E)	Mid (mean ± S.E)	p value	Final (mean ± S.E)	p value
Total-C (mmol/L)	5.40±0.22	5.87 ± 0.21	0.09	5.55 ± 0.24	0.33
HDL-C (mmol/L)	1.45± 0.16	1.28±0.16	0.04	1.13±0.12	0.02
LDL-C (mmol/L)	3.11 ± 0.19	3.57 ± 0.22	0.07	3.69 ± 0.26	0.04
Lp(a) (nmol/L)	69.63 ± 33.53	73.73 ± 30.25	0.33	65.07 ± 28.93	0.27
TG (mmol/L)	1.79 ± 0.29	2.18 ± 0.27	0.19	1.58 ± 0.24	0.11
ApoA1 (g/L)	1.81 ± 0.10	1.78 ± 0.12	0.40	1.63 ± 0.09	0.05
ApoB (g/L)	0.97 ± 0.05	1.05 ± 0.05	0.07	1.12 ± 0.06	0.001

*Note*: Total-C = Total cholesterol; HDL-C = High density lipoprotein cholesterol; LDL-C = Low density lipoprotein cholesterol; Lp(a) = Lipoprotein(a); TG = Triglycerides; ApoA1 = Apolipoprotein A1; ApoB = ApolipoproteinB-100.

p values were calculated using paired student t-test.

### Doxorubicin reduces the expression of ABCA1 and apoA1 in HepG2 cells

**Fig 2:**
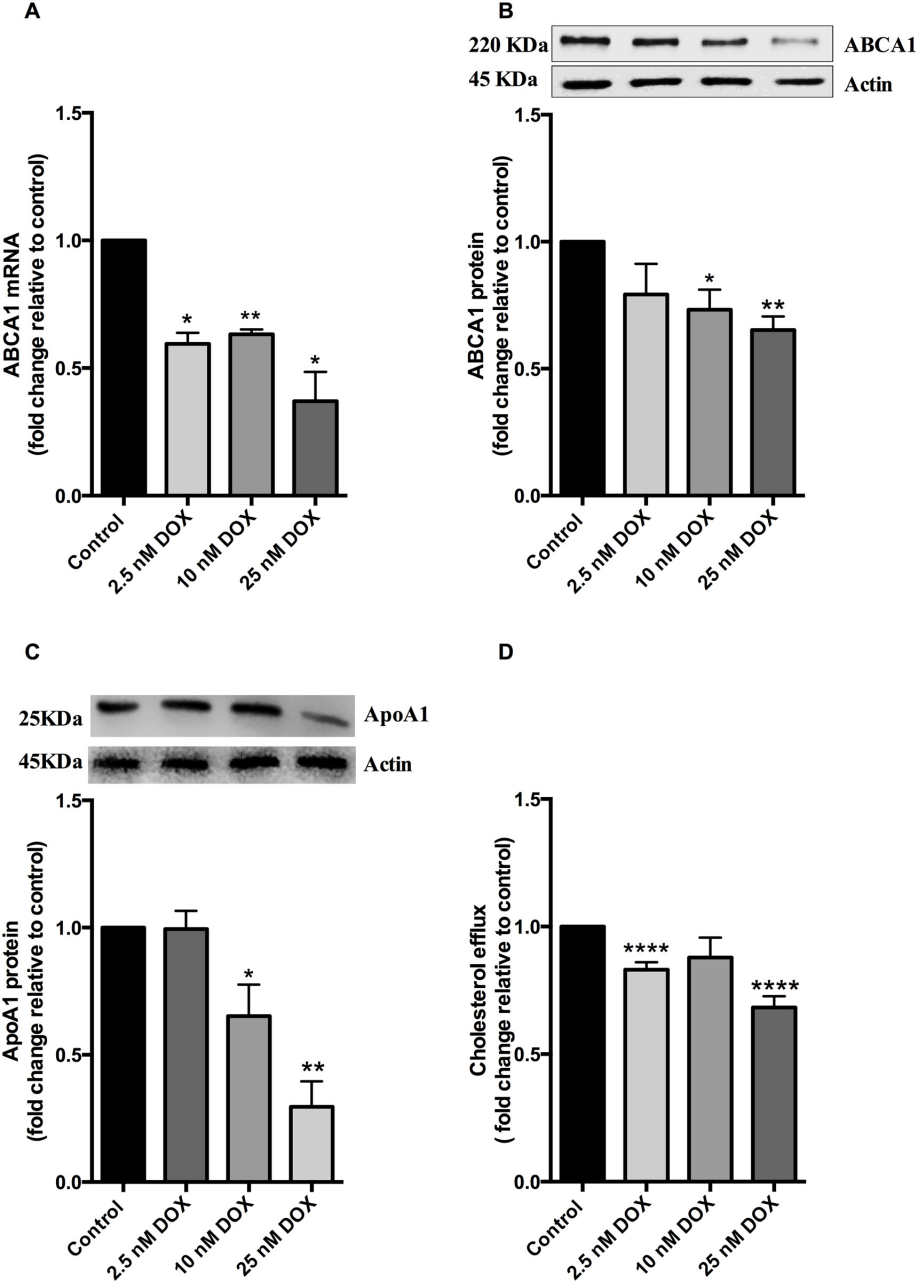
Doxorubicin reduces ABCA1 and apoA1 levels in HepG2 cells. HepG2 cells were treated with 2.5 nM, 10 nM and 25 nM doxorubicin (DOX) for 24 hours at 37°C. ABCA1 mRNA levels (A) were quantified by RT-PCR after normalising against β2-microglobulin and RPL27 mRNA. ABCA1 protein levels (B) and apoA1 protein levels (C) were quantified by western blotting after normalising to actin (see inset). All results are expressed relative to that of the untreated control. Cholesterol efflux to apoA1 was also measured after doxorubicin treatment (D). HepG2 cells were loaded with [^3^H] cholesterol for 48 hours prior to treatment then incubated with apoA1 acceptor for 2 hours after treatment and apoA1-mediated efflux calculated. Results are expressed as mean ± S.E for three experiments performed in triplicates for RT-PCR and at least two experiments performed in duplicate for protein quantification. Cholesterol efflux assays were performed in triplicate. *, p< 0.05 **, p< 0.01 ***, p< 0.001 compared with untreated control.

Since the expression of ABCA1 in liver cells is a major contributor to HDL levels via its role in cholesterol efflux [19], we investigated whether the chemotherapy agents used in the treatment regime of the patients affected ABCA1 levels in hepatocytes. Treatment of HepG2 cells with doxorubicin at a range of physiological concentrations caused a significant reduction in ABCA1 mRNA transcript levels down to 0.35 fold at 25 nM (Fig 2A). This was accompanied by a concentration dependent decrease in ABCA1 protein levels down to 0.65 fold at 25 nM doxorubicin (Fig 2B). Doxorubicin also decreased apoA1 protein levels to 0.65 fold and 0.30 fold at 10 nM and 25 nM doxorubicin (Fig 2C). The suppression of the ABCA1 pathway was also evident at a functional level with doxorubicin treatment significantly decreasing apoA1-mediated cholesterol efflux by 20% at 2.5 nM and 30% at 25 nM (Fig 2D). The effect of epirubicin on ABCA1 was also tested, as some patients received epirubicin instead of doxorubicin. Epirubicin decreased ABCA1 protein expression to a similar extent to doxorubicin (0.60 fold at 10 nM and 25 nM, S2 Fig).

### Doxorubicin reduces the expression of PPARγ and LXRα genes in HepG2 cells

**Fig 3:**
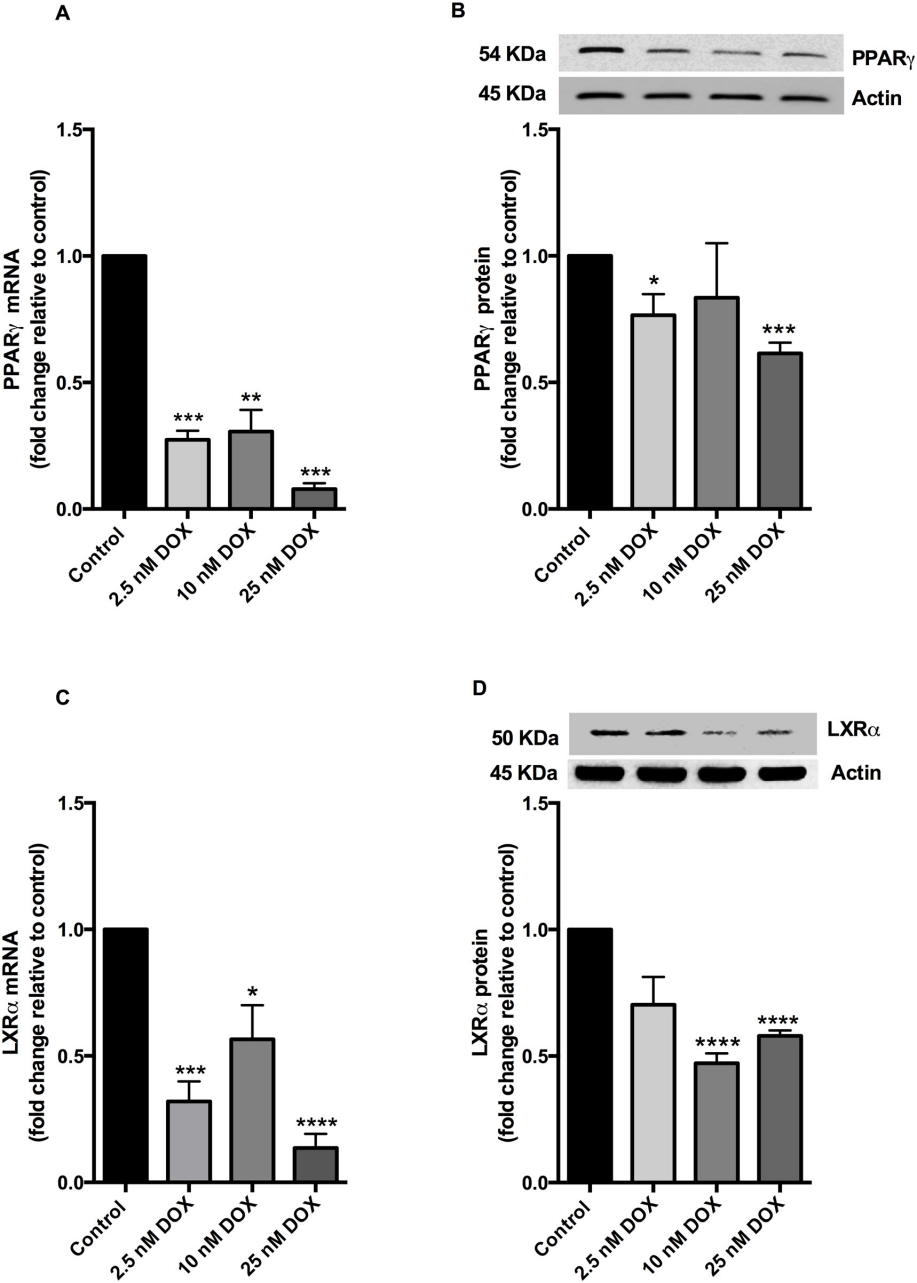
Doxorubicin reduces PPARγ and LXRα levels in HepG2 cells. HepG2 cells were treated with 2.5 nM, 10 nM and 25 nM doxorubicin (DOX) for 24 hours at 37°C. mRNA and protein levels of PPARγ (A, B) and LXRα (C, D) were determined. mRNA levels were quantified by RT-PCR after normalising against β2-microglobulin and RPL27 mRNA. Protein levels were quantified by western blotting after normalising to actin (see inset). All results are expressed relative to that of the untreated control. Results are expressed as mean ± S.E for three experiments performed in triplicates for RT-PCR and at least two experiments performed in duplicate for protein quantification. *, p< 0.05 **, p< 0.01 ***, p< 0.001 compared with untreated control.

Since the nuclear receptor PPARγ regulates ABCA1 expression by LXRα [20], we checked the effect of doxorubicin on these. Doxorubicin significantly reduced PPARγ mRNA (by 0.27 fold at 2.5 nM down to 0.08 fold at 25 nM) (Fig 3A) and protein levels (by 0.76 fold at 2.5 nM down to 0.60 fold at 25 nM (Fig 3B). A significant decrease was also apparent in LXRα mRNA (down to 0.13 fold at 25 nM) (Fig 3C) and protein levels (reduced to 0.6 fold at 25 nM doxorubicin) (Fig 3D). With respect to other targets of PPARγ and LXRα being modulated by doxorubicin, RXRα was investigated as a target for PPARγ and was also shown to be decreased by doxorubicin (data not shown).

### Cyclophosphamide and paclitaxel have no effect on ABCA1 and apoA1 in HepG2 cells

**Fig 4:**
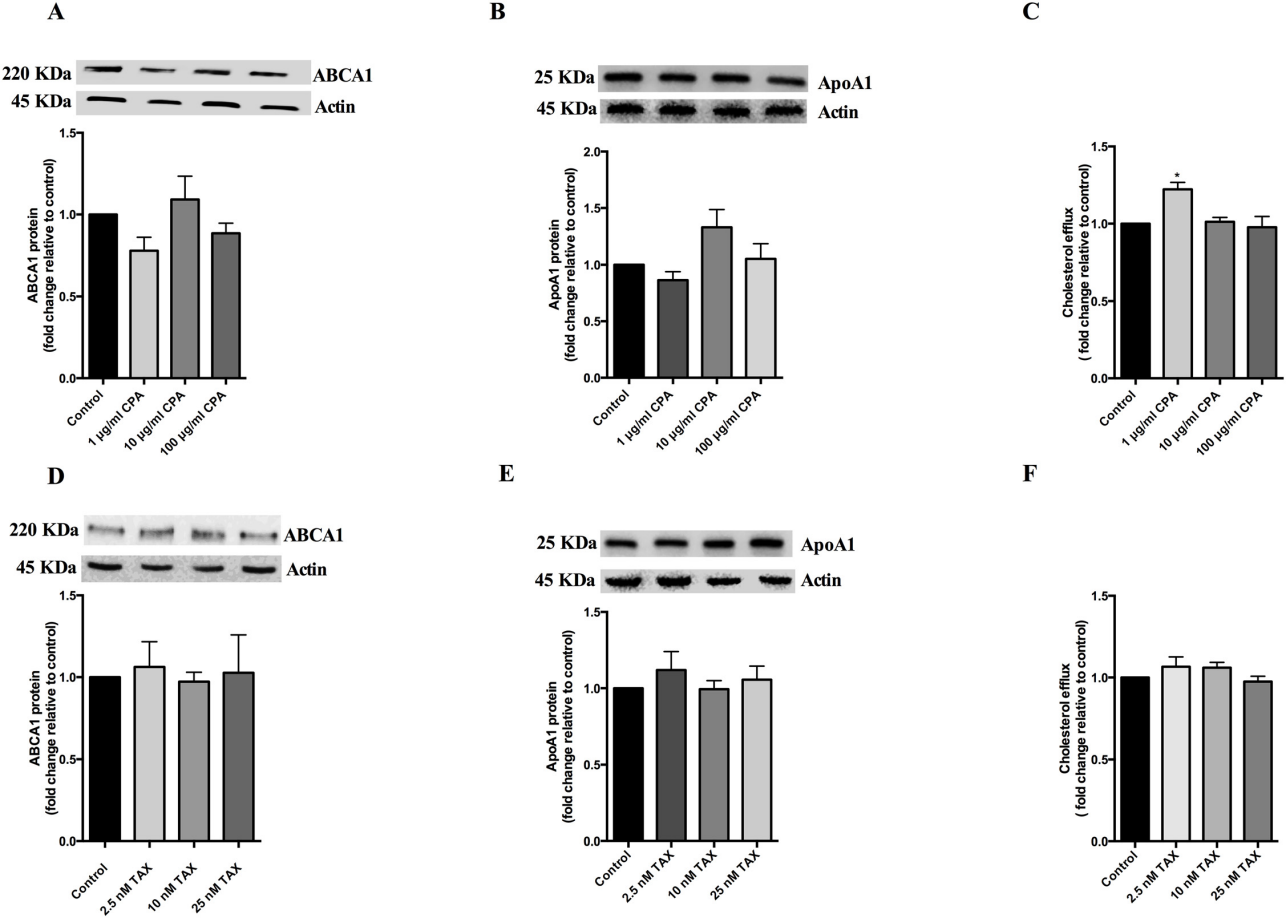
Cyclophosphamide and Paclitaxel did not affect ABCA1 or apoA1 levels. HepG2 cells were treated with 1 μg/ml, 10 μg/ml and 100 μg/ml cyclophosphamide (CPA) or 2.5 nM, 10 nM and 25 nM paclitaxel (TAX) for 24 hours at 37°C. ABCA1 and apoA1 protein levels after cyclophosphamide (A, B) and paclitaxel (D, E) treatment were determined by western blotting and expressed relative to that of untreated cells after normalisation against actin (see inset). HepG2 cells were loaded with [^3^H] cholesterol for 48 hours prior to treatment and cells were incubated with apoA1 acceptor after treatment for 2 hours. ApoA1 mediated cholesterol efflux was calculated after cyclophosphamide (C) and paclitaxel (F) treatment. Results are expressed as two experiments performed in triplicate for protein quantification and triplicate experiments for cholesterol efflux assays. *, p< 0.05 **, p< 0.01 ***, p< 0.001 compared with untreated control.

As the breast cancer patients were on combined doxorubicin (or epirubicin) and cyclophosphamide therapy for 12 weeks followed by a further 12 weeks on paclitaxel (or docetaxel), we also investigated the effect of physiological levels of cyclophosphamide and paclitaxel on ABCA1. Cyclophosphamide and paclitaxel did not appear to significantly affect ABCA1 protein levels (Fig 4A and 4D) or apoA1-mediated cholesterol efflux in HepG2 cells (Fig 4C and 4F) apart from increase in efflux at the lowest concentration of cyclophosphamide which was not reproduced at higher concentrations (Fig 4C). Additionally, cyclophosphamide and paclitaxel did not alter apoA1 protein levels (Fig 4B and 4E).

### Doxorubicin and paclitaxel increase apoB protein levels in HepG2 cells

**Fig 5:**
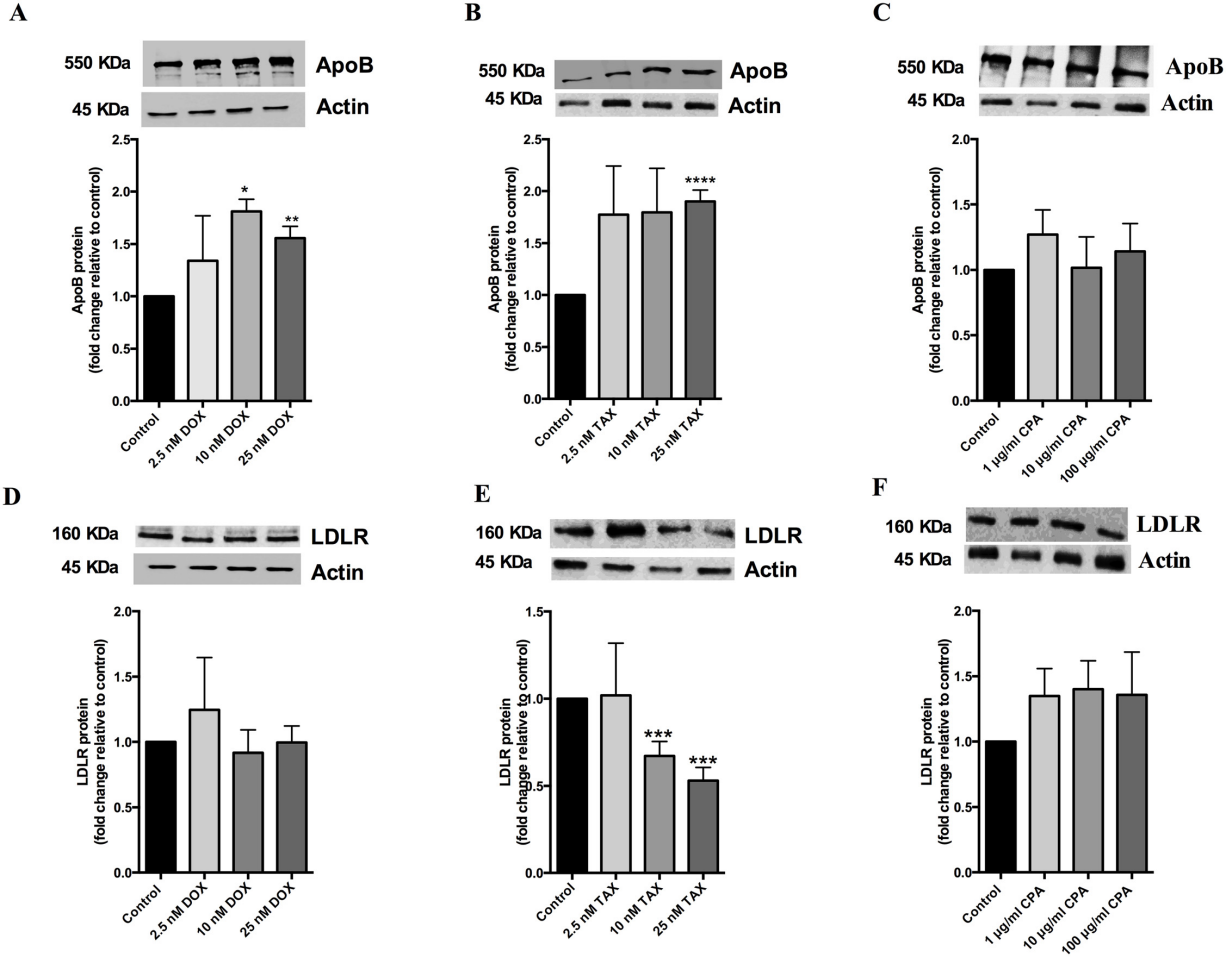
Doxorubicin and paclitaxel increase apoB levels whereas cyclophosphamide did not affect apoB levels in HepG2 cells. HepG2 cells were treated with of doxorubicin (DOX) or paclitaxel (TAX) at 2.5 nM, 10 nM and 25 nM concentration or 1 μg/ml, 10 μg/ml and 100 μg/ml cyclophosphamide (CPA) for 24 hours at 37°C. ApoB protein (A, B, C) and LDLR protein (D, E, F) levels were determined after treatment by western blot after normalizing against actin (see inset). Protein levels are expressed relative to that of untreated control cells. Results are expressed as mean ± S.E for two experiments performed in triplicate for western blots. *, p< 0.05 **, p< 0.01 ***, p< 0.001 compared with control.

As chemotherapy treatment significantly increased apoB levels in breast cancer patients, we investigated the effect of doxorubicin, cyclophosphamide and paclitaxel on apoB protein levels in HepG2 cells. Doxorubicin and paclitaxel increased the apoB protein level by 1.6 fold and by 1.9 fold at 25 nM, respectively (Fig 5A and 5B). Whereas, cyclophosphamide did not affect apoB protein levels (Fig 5C). As the LDLR is responsible for clearance of apoB-containing particles, we also checked whether either treatment affected LDLR protein levels. The LDLR protein levels were unchanged by doxorubicin and cyclophosphamide treatment (Fig 5D and 5F), but paclitaxel significantly decreased LDLR protein levels 0.67 fold and 0.53 fold at 10 nM and 25 nM, respectively (Fig 5E).

### Doxorubicin decreases and paclitaxel increases HMG-CoA reductase levels in HepG2 cells

We investigated the effects of doxorubicin, cyclophosphamide and paclitaxel on 3-Hydroxy-3-Methylglutaryl-CoA Reductase (HMGCR), the rate controlling enzyme required for cholesterol synthesis (25). Doxorubicin significantly reduced HMGCR protein levels to 0.45 fold at 25 nM doxorubicin (S1A Fig). In contrast, paclitaxel significantly increased HMGCR protein levels by 1.5 fold at 25 nM paclitaxel (S1C Fig), while cyclophosphamide did not alter HMGCR protein levels (S1B Fig).

## Discussion

Our results show that chemotherapy significantly alters plasma lipid and apolipoprotein levels in breast cancer patients. Specifically, HDL-C and apoA1 levels were significantly decreased and apoB levels significantly increased. Lipid modulations did not appear to be affected by the disease severity. Reduced HDL-C and apoA1 levels and increased apoB levels are well established risk factors for the development of CVD [21]. Several studies have previously reported alterations in lipid levels in cancer patients undergoing chemotherapy, but the magnitude and direction of change reported, varied [12, 13, 22]. These observations, and those from the current study, are based on a limited number of subjects, but nonetheless constitute evidence to suggest that lipid metabolism is altered in individuals undergoing chemotherapy. Cancer patients are often given a combination of chemotherapy agents for different lengths of time, as seen here, making it difficult to dissect the effect of any one agent. For this reason we included an *in vitro* study to look at the effect of each chemotherapy agent in isolation. We hypothesised that different agents would have different effects on lipid metabolism genes in the liver, which may relate to the changes seen in plasma lipid profiles. We show in an *in vitro* setting that the different agents used in combination chemotherapy do have differing effects on genes involved in lipid metabolism. As far as we are aware, our study is the first to dissect the effect of various chemotherapy agents on lipid metabolism at a cellular level. Interestingly, these effects were reflected in the observed changes in the lipid profiles of the patients.

We showed that doxorubicin reduced the expression of the ABCA1 gene in liver cells through downregulation of the LXRα and PPARγ nuclear receptors that regulate its expression. Moreover, doxorubicin decreased apoA1 protein levels as well. This effect appeared to be specific to doxorubicin as no effect on ABCA1 and apoA1 levels was apparent from cyclophosphamide or paclitaxel. As ABCA1 and apoA1 are crucial for HDL production from the liver [19], this effect most likely explains the association of doxorubicin with reduced HDL levels as seen here. Low HDL-C levels have also been reported in another study of breast cancer patients on doxorubicin therapy [23]; however, baseline levels were not measured in that study. Based on patient HDL-C levels in our study, it appeared that the effect of doxorubicin extended beyond the treatment period since HDL-C levels were still reduced 9–12 weeks after the last doxorubicin treatment. During this time patients were treated with paclitaxel alone, and based on our cellular studies, it was expected that paclitaxel would have no effect on HDL-C due to its lack of an effect on ABCA1. The effect of cumulative doses of doxorubicin on HDL-C levels and an evaluation of the time course of return to baseline levels after treatment will be required before the likely effect on CVD risk can be predicted.

Doxorubicin (and epirubicin) are anthracyclines that intercalate between DNA to inhibit both DNA and RNA synthesis. One would therefore expect a global reduction in gene expression with doxorubicin treatment. While this seems to be the case for genes in the HDL production pathway, it is not always so. Doxorubicin treatment is known to increase the expression of some inflammatory genes [24] and here we also show that doxorubicin increases the expression of apoB in liver cells.

A significant increase in apoB levels was seen in patients at the final time point after single agent paclitaxel treatment. Interestingly, our cellular studies showed that the taxane, paclitaxel, promoted an increase in apoB expression (more so than doxorubicin) and in addition induced a significant reduction in LDLR expression. Overexpression of apoB in the liver would be expected to increase the secretion of apoB-associated lipoproteins into blood and a reduction in LDLR, the main removal route for LDL, would also be expected to increase apoB levels. These combined effects likely explain why the apoB increase was only significant after paclitaxel treatment. The length of effect of paclitaxel on apoB levels after completion of treatment remains to be seen.

Chemotherapy drugs also showed a differential effect on HMGCR, the rate determining enzyme required for cholesterol synthesis [25]. Doxorubicin decreased HMGCR protein levels whereas paclitaxel increased HMGCR protein levels, while no effect was seen with cyclophosphamide suggesting a variable response of chemotherapy drugs on cholesterol synthesis.

In conclusion, our study shows that lipid alterations occur with chemotherapy and are specific to the chemotherapy agent used. Doxorubicin promotes an HDL-C lowering effect while paclitaxel promotes an increase in apoB, the combination of which would be expected to have negative consequences on CVD risk. In contrast, cyclophosphamide does not seem to perturb HDL or apoB metabolism. Although the longevity of the lipid alterations needs to be established, our study suggests that lipid alterations should be considered in the longer term management plan for cancer patients in order to reduce their risk of CVD.

### Limitations of the study

The lack of follow up lipid data is the major limitation to the study and would be required to establish if the lipid changes were maintained and therefore could have clinical implications. The study also only investigated a limited number of lipid metabolism targets in response to chemotherapy drug treatment. It would be useful to investigate a broader range of metabolic targets. It would also be useful to compare other classes of chemotherapeutic drugs such as the antiestrogenic and immunomodulating drugs for their effect on lipid levels. Furthermore, the exact biological actions of chemotherapy agents need to be better characterised.

## Supporting Information

S1 Fig Doxorubicin reduces and paclitaxel increases HMGCR levels in HepG2 cells. (DOCX)

S2 Fig Epirubicin reduces ABCA1 protein levels in HepG2 cells. (DOCX)

S1 Table Lipid levels of individual breast cancer patients at baseline. (DOCX)

S2 Table Sequences of gene specific primers used for RT-PCR. (DOCX)

## References

[1] Jemal A, Bray F, Center MM, Ferlay J, Ward E, Forman D. Global cancer statistics. CA: A Cancer Journal for Clinicians. 2011;61: 69–90. 21296855 10.3322/caac.20107

[2] Early Breast Cancer Trialists’ Collaborative Group (EBCTCG). Effects of chemotherapy and hormonal therapy for early breast cancer on recurrence and 15-year survival: an overview of the randomised trials. Lancet. 2005;365: 1687–717. 15894097 10.1016/S0140-6736(05)66544-0

[3] Schultz PN, Beck ML, Stava C, Vassilopoulou-Sellin R. Health profiles in 5836 long-term cancer survivors. Int J Cancer J Int Cancer. 2003;104: 488–95. 10.1002/ijc.10981 12584748

[4] Pein F, Sakiroglu O, Dahan M, Lebidois J, Merlet P, Shamsaldin A, et al Cardiac abnormalities 15 years and more after adriamycin therapy in 229 childhood survivors of a solid tumour at the Institut Gustave Roussy. Br J Cancer. 2004;91: 37–44. 15162142 10.1038/sj.bjc.6601904 PMC2364747

[5] Shenoy C, Klem I, Crowley AL, Patel MR, Winchester MA, Owusu C, et al Cardiovascular Complications of Breast Cancer Therapy in Older Adults. The Oncologist. 2011;16: 1138–43. 10.1634/theoncologist.2010-0348 21737575 PMC3228150

[6] Patnaik JL, Byers T, DiGuiseppi C, Dabelea D, Denberg TD. Cardiovascular disease competes with breast cancer as the leading cause of death for older females diagnosed with breast cancer: a retrospective cohort study. Breast Cancer Res. 2011;13: R64 10.1186/bcr2901 21689398 PMC3218953

[7] Mamounas EP, Bryant J, Lembersky B, Fehrenbacher L, Sedlacek SM, Fisher B, et al Paclitaxel After Doxorubicin Plus Cyclophosphamide As Adjuvant Chemotherapy for Node-Positive Breast Cancer: Results From NSABP B-28. J Clin Oncol. 2005;23: 3686–96. 15897552 10.1200/JCO.2005.10.517

[8] Octavia Y, Tocchetti CG, Gabrielson KL, Janssens S, Crijns HJ, Moens AL. Doxorubicin-induced cardiomyopathy: From molecular mechanisms to therapeutic strategies. J Mol Cell Cardiol. 2012;52: 1213–25. 10.1016/j.yjmcc.2012.03.006 22465037

[9] Steinherz LJ, Steinherz PG, Mangiacasale D, O’Reilly R, Allen J, Sorell M, et al Cardiac changes with cyclophosphamide. Med Pediatr Oncol. 1981;9: 417–22. 7300803 10.1002/mpo.2950090502

[10] Gianni L, Munzone E, Capri G, Fulfaro F, Tarenzi E, Villani F, et al Paclitaxel by 3-hour infusion in combination with bolus doxorubicin in women with untreated metastatic breast cancer: high antitumor efficacy and cardiac effects in a dose-finding and sequence-finding study. J Clin Oncol Off J Am Soc Clin Oncol. 1995;13: 2688–99. 10.1200/JCO.1995.13.11.2688 7595726

[11] Gollerkeri A, Harrold L, Rose M, Jain D, Burtness BA. Use of paclitaxel in patients with pre-existing cardiomyopathy: a review of our experience. Int J Cancer J Int Cancer. 2001;93: 139–41. 10.1002/ijc.1295 11391633

[12] Alexopoulos CG, Pournaras S, Vaslamatzis M, Avgerinos A, Raptis S. Changes in serum lipids and lipoproteins in cancer patients during chemotherapy. Cancer Chemother Pharmacol. 1992;30: 412–6. 1505080 10.1007/BF00689971

[13] Rzymowska J. Effect of cytotoxic chemotherapy on serum lipid levels in breast cancer patients. Pathobiol J Immunopathol Mol Cell Biol. 1999;67: 129–32. 10.1159/000028062 10394133

[14] Friedewald WT, Levy RI, Fredrickson DS. Estimation of the concentration of low-density lipoprotein cholesterol in plasma, without use of the preparative ultracentrifuge. Clin Chem. 1972;18: 499–502. 4337382

[15] Marcovina SM, Albers JJ, Gabel B, Koschinsky ML, Gaur VP. Effect of the number of apolipoprotein(a) kringle 4 domains on immunochemical measurements of lipoprotein(a). Clin Chem. 1995;41: 246–55. 7533064

[16] Pease RJ, Milne RW, Jessup WK, Law A, Provost P, Fruchart JC, et al Use of bacterial expression cloning to localize the epitopes for a series of monoclonal antibodies against apolipoprotein B100. J Biol Chem. 1990;265: 553–68. 1688435

[17] Strober W. Trypan blue exclusion test of cell viability. Curr Protoc Immunol Ed John E Coligan Al. 2001; Appendix 3:Appendix 3B. 10.1002/0471142735.ima03bs21 18432654

[18] Brace RJ, Sorrenson B, Sviridov D, McCormick SPA. A gel-based method for purification of apolipoprotein A-I from small volumes of plasma. J Lipid Res. 2010;51: 3370–6. 10.1194/jlr.D008300 20667818 PMC2952579

[19] Basso F, Freeman L, Knapper CL, Remaley A, Stonik J, Neufeld EB, et al Role of the hepatic ABCA1 transporter in modulating intrahepatic cholesterol and plasma HDL cholesterol concentrations. J Lipid Res. 2003;44: 296–302. 12576511 10.1194/jlr.M200414-JLR200

[20] Boisvert WA, Lee C-H, Laffitte BA, Barak Y, Joseph SB, Liao D, et al A PPARγ-LXR-ABCA1 Pathway in Macrophages Is Involved in Cholesterol Efflux and Atherogenesis. Mol Cell. 2001;7: 161–71. 11172721 10.1016/s1097-2765(01)00164-2

[21] Schaefer EJ, Lamon-Fava S, Cohn SD, Schaefer MM, Ordovas JM, Castelli WP, et al Effects of age, gender, and menopausal status on plasma low density lipoprotein cholesterol and apolipoprotein B levels in the Framingham Offspring Study. J Lipid Res. 1994;35: 779–92. 8071601

[22] Raghavan D, Cox K, Childs A, Grygiel J, Sullivan D. Hypercholesterolemia after chemotherapy for testis cancer. J Clin Oncol Off J Am Soc Clin Oncol. 1992;10: 1386–9. 10.1200/JCO.1992.10.9.1386 1325540

[23] Bell KE, Di Sebastiano KM, Vance V, Hanning R, Mitchell A, Quadrilatero J, et al A comprehensive metabolic evaluation reveals impaired glucose metabolism and dyslipidemia in breast cancer patients early in the disease trajectory. Clin Nutr. 2014;33: 550–7. 10.1016/j.clnu.2013.08.001 24011971

[24] Sauter KA, Wood LJ, Wong J, Iordanov M, Magun BE. Doxorubicin and daunorubicin induce processing and release of interleukin-1β through activation of the NLRP3 inflammasome. Cancer Biol Ther. 2011;11: 1008–16. 21464611 10.4161/cbt.11.12.15540 PMC3142364

[25] Sharpe LJ, Brown AJ. Controlling cholesterol synthesis beyond 3-hydroxy-3- methylglutaryl-CoA reductase (HMGCR). J Biol Chem. 2013;288: 18707–15. 10.1074/jbc.R113.479808 23696639 PMC3696645

