# Supplementary material for: Chemotherapy Agents Alter Plasma Lipids in Breast Cancer Patients and Show Differential Effects on Lipid Metabolism Genes in Liver Cells
Source: PLoS One. 2016 Jan 25;11(1):e0148049. doi: 10.1371/journal.pone.0148049 (PMC4726544; doi:10.1371/journal.pone.0148049)

**S1 Fig. Doxorubicin reduces and paclitaxel increases HMGCR levels in HepG2 cells.** HepG2 cells were treated with doxorubicin (DOX) or paclitaxel (TAX) at 2.5 nM, 10 nM and 25 nM or 1 µg/ml, 10 µg/ml and 100 µg/ml cyclophosphamide (CPA) for 24 hours at 37^º^C. HMGCR protein levels (A, B and C) were determined after treatment by western blot after normalizing against actin (see inset). Protein levels are expressed relative to that of untreated control cells. Results are expressed as mean ± S.E for triplicate western blots. *, p< 0.05 **, p< 0.01 ***, p< 0.001 compared with control.


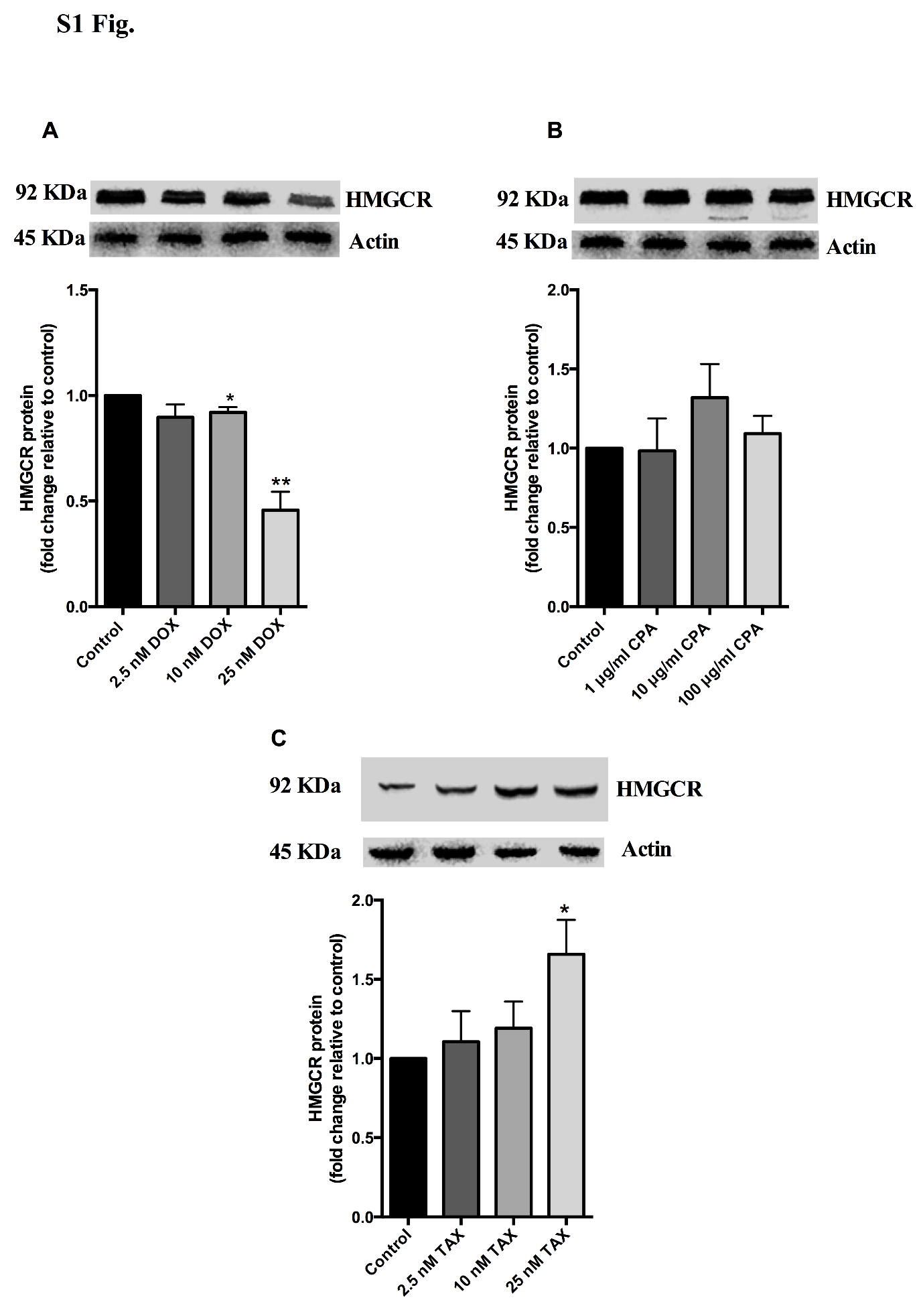

Supplement: S1 Fig — (DOCX) [file pone.0148049.s001.docx]
