# Supplementary material for: Chemotherapy Agents Alter Plasma Lipids in Breast Cancer Patients and Show Differential Effects on Lipid Metabolism Genes in Liver Cells
Source: PLoS One. 2016 Jan 25;11(1):e0148049. doi: 10.1371/journal.pone.0148049 (PMC4726544; doi:10.1371/journal.pone.0148049)

**S2 Fig. Epirubicin reduces ABCA1 protein levels in HepG2 cells.** HepG2 cells were treated with 2.5 nM, 10 nM and 25 nM of epirubicin (EPI) for 24 hours at 37ºC. ABCA1 protein levels were determined after treatment by western blot after normalizing against actin. Protein levels are expressed relative to that of untreated control cells. Results are expressed as mean ± S.E for two experiments performed in triplicate for western blots.*, p< 0.05 **, p< 0.01 ***, p< 0.001 compared with control.


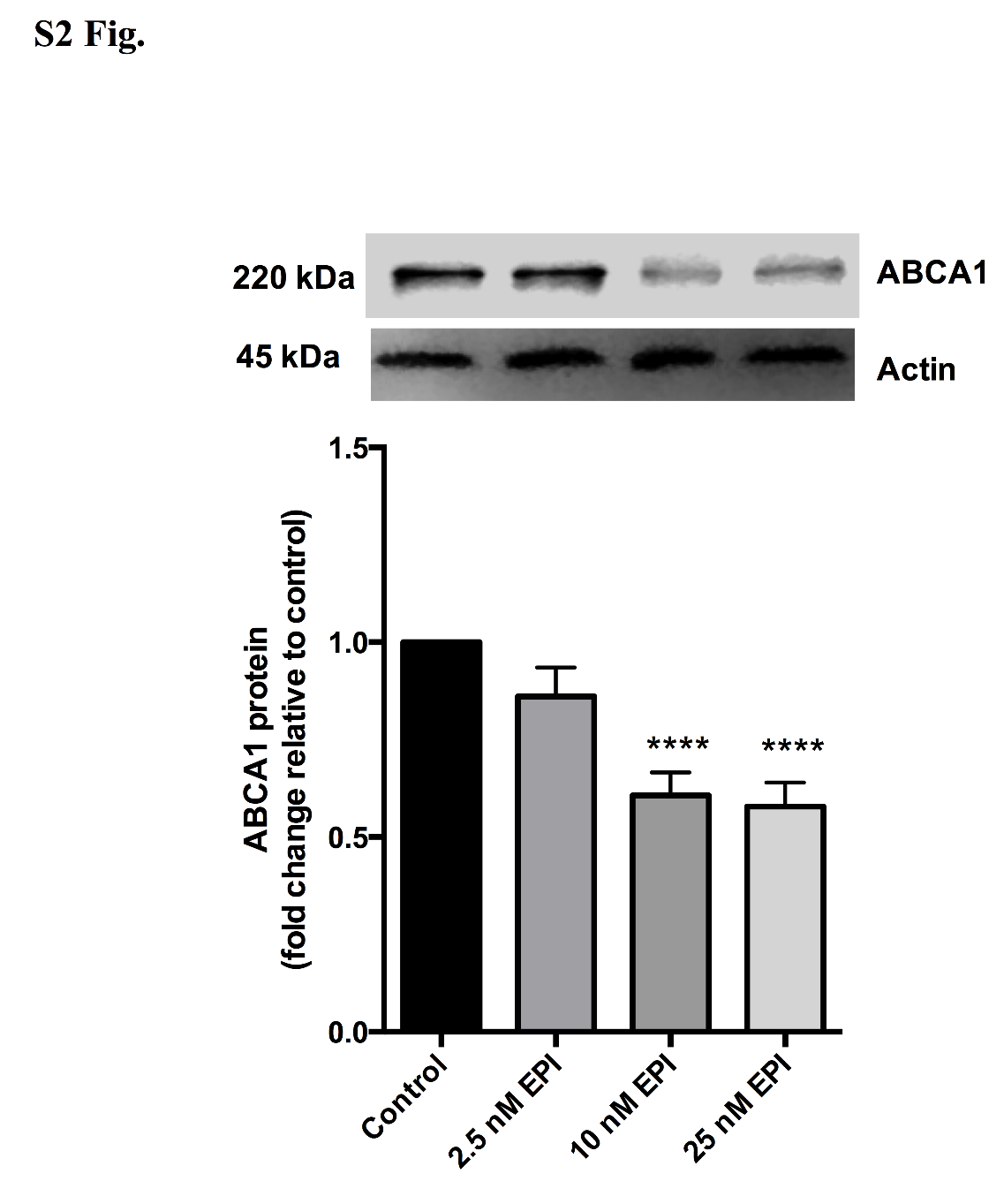

Supplement: S2 Fig — (DOCX) [file pone.0148049.s002.docx]
