# Supplementary material for: Chemotherapy Agents Alter Plasma Lipids in Breast Cancer Patients and Show Differential Effects on Lipid Metabolism Genes in Liver Cells
Source: PLoS One. 2016 Jan 25;11(1):e0148049. doi: 10.1371/journal.pone.0148049 (PMC4726544; doi:10.1371/journal.pone.0148049)
Supplement: S1 Table — (DOCX) [file pone.0148049.s003.docx]

**S1 Table: Lipid levels of individual breast cancer patients at baseline.**

| Breast Cancer Patients (BCP) | | | | | | | | | | | | |
| --- | --- | --- | --- | --- | --- | --- | --- | --- | --- | --- | --- | --- |
|  | A | B | C | D | E | F | G | H | I | J | K | L |
| Total-C (mmol/L) | 4.49 | 4.98 | 6.41 | 5.17 | 6.44 | 4.16 | 6.28 | 5.43 | 6.04 | 4.4 | 5.65 | 5.42 |
| HDL-C (mmol/L) | 1.32 | 1.35 | 2.82 | 1.98 | 1.44 | 0.78 | 1.35 | 0.87 | 1.44 | 1.11 | 1.17 | 1.74 |
| LDL-C (mmol/L) | 2.73 | 2.52 | 3.13 | 1.87 | 4.50 | 3.10 | 3.32 | 3.40 | 3.07 | 2.52 | 3.90 | 3.33 |
| Lp(a) (nmol/L) | 4.7 | 4.1 | 10.95 | 6.65 | 5.17 | 8.52 | 72.3 | 384.7 | 12.2 | 204.1 | 104.4 | 17.7 |
| TG (mmol/L) | 0.95 | 1.96 | 1.01 | 2.88 | 1.08 | 0.62 | 3.51 | 2.52 | 3.33 | 1.67 | 1.26 | 0.75 |
| ApoA1 (g/L) | 1.97 | 2.0 | 2.3 | 2.24 | 1.86 | 1.41 | 1.44 | 1.2 | 2.18 | 1.53 | 1.72 | 1.95 |
| ApoB (mg/dL) | 103 | 106 | 95 | 66 | 95 | 110 | 81 | 90 | 128 | 124 | 92 | 77 |

*Note:* Total-C = Total cholesterol; HDL-C = High density lipoprotein cholesterol; LDL-C = Low density lipoprotein cholesterol; Lp(a) = Lipoprotein(a); TG = Triglycerides; ApoA1 = Apolipoprotein A1; ApoB = ApolipoproteinB-100.
