# Supplementary material for: Chemotherapy Agents Alter Plasma Lipids in Breast Cancer Patients and Show Differential Effects on Lipid Metabolism Genes in Liver Cells
Source: PLoS One. 2016 Jan 25;11(1):e0148049. doi: 10.1371/journal.pone.0148049 (PMC4726544; doi:10.1371/journal.pone.0148049)
Supplement: S2 Table — (DOCX) [file pone.0148049.s004.docx]

**S2 Table: Sequences of gene specific primers used for RT-PCR.**

| Gene | Forward Primer (5’-3’) | Reverse Primer (5’-3’) |
| --- | --- | --- |
| ABCA1 | GGTAGGAGAAAGAGACGCAAAC | AACAAAACAATAACGCCCAAGT |
| β-2M | TTTCATCCATCCGACATTGA | CCTCCATGATGCTGCTTACA |
| LXRα | CGGGCTTCCACTACAATGTT | TCAGGCGGATCTGTTCTTCT |
| PPARγ | GTGTTGGTGATAGGTCCGAAAT | CCCAAGTGAATTGGATTCTTCT |
| RPL27 | ATCGCCAAGAGATCAAAGATAA | TCTGAAGACATCCTTATTGACG |
